# Efficacy and safety of tafolecimab in Chinese patients with heterozygous familial hypercholesterolemia: a randomized, double-blind, placebo-controlled phase 3 trial (CREDIT-2)

**DOI:** 10.1186/s12916-023-02797-8

**Published:** 2023-02-28

**Authors:** Meng Chai, Yongming He, Wang Zhao, Xuebin Han, Guoyan Zhao, Xueping Ma, Ping Qiao, Dongmei Shi, Yuyang Liu, Wei Han, Pei An, Haoyu Li, Shuling Yan, Qingyang Ma, Huan Deng, Lei Qian, Yujie Zhou, on behalf of CREDIT-2 investigators

**Affiliations:** 1 Department of Cardiology, Beijing Anzhen Hospital, Capital Medical University, Beijing Institute of Heart Lung and Blood Vessel Disease, Beijing Key Laboratory of Precision Medicine of Coronary Atherosclerotic Disease, Clinical Center for Coronary Heart Disease, Capital Medical University, No.2 Anzhen Road, ChaoYang District, Beijing, 100029 China; 2 Division of Cardiology, The First Affiliated Hospital of Soochow University, Suzhou, China; 3 Department of Cardiovascular Medicine, The Second Xiangya Hospital, Central South University, Changsha, China; 4 Department of Cardiology, Shanxi Cardiovascular Hospital, Taiyuan, China; 5 Department of Cardiovascular Disease, Tianjin Medical University General Hospital, Tianjin, China; 6 Department of Cardiovascular Medicine, General Hospital of Ningxia Medical University, Yinchuan, China; 7 Department of Cardiology, Hainan Provincial People’s Hospital, Haikou, China; 8 Innovent Biologics, Inc., Suzhou, China

**Keywords:** Tafolecimab, PCSK9, LDL-C, HeFH

## Abstract

**Background:**

Heterozygous familial hypercholesterolemia (HeFH) is largely underdiagnosed and undertreated in China where few patients achieved recommended target levels of low density lipoprotein cholesterol (LDL-C). We conducted the first randomized, placebo-controlled clinical trial in Chinese patients with HeFH to assess the efficacy and safety of tafolecimab, a novel fully human proprotein convertase subtilisin/kexin type 9 (PCSK9) monoclonal antibody.

**Methods:**

Patients diagnosed with HeFH by Simon Broome criteria and on a stable lipid-lowering therapy for at least 4 weeks were randomized 2:2:1:1 to receive subcutaneous tafolecimab 150 mg every 2 weeks (Q2W), tafolecimab 450 mg every 4 weeks (Q4W), placebo Q2W or placebo Q4W in the 12-week double-blind treatment period. After that, participants received open-label tafolecimab 150 mg Q2W or 450 mg Q4W for 12 weeks. The primary endpoint was the percent change from baseline to week 12 in LDL-C levels. Secondary endpoints included proportion of participants achieving ≥50% LDL-C reductions and proportion of participants with LDL-C <1.8 mmol/L at week 12 and 24, the change from baseline to week 12 in non-high density lipoprotein cholesterol (non-HDL-C), apolipoprotein B and lipoprotein(a) levels, as well as the change from baseline to week 24 in lipid levels.

**Results:**

In total, 149 participants were randomized and 148 received at least one dose of the study treatment. At week 12, tafolecimab treatment induced significant reductions in LDL-C levels (treatment difference versus placebo [on-treatment estimand]: −57.4% [97.5% CI, −69.2 to −45.5] for 150 mg Q2W; −61.9% [−73.4 to −50.4] for 450 mg Q4W; both *P* <0.0001). At both dose regimens, significantly more participants treated with tafolecimab achieved ≥50% LDL-C reductions or LDL-C <1.8 mmol/L at week 12 as compared with corresponding placebo groups (all *P* <0.0001). Meanwhile, non-HDL-C, apolipoprotein B and lipoprotein(a) levels were significantly reduced in the tafolecimab groups at week 12. The lipid-lowering effects of tafolecimab were maintained till week 24. During the double-blind treatment period, the most commonly-reported adverse events in the tafolecimab groups included upper respiratory tract infection, increased blood creatine phosphokinase, increased alanine aminotransferase, increased aspartate aminotransferase and hypertension.

**Conclusions:**

Tafolecimab administered either 150 mg Q2W or 450 mg Q4W yielded significant and persistent reductions in LDL-C levels and showed a favorable safety profile in Chinese patients with HeFH.

**Trial registration:**

ClinicalTrials.gov, NCT04179669.

**Supplementary Information:**

The online version contains supplementary material available at 10.1186/s12916-023-02797-8.

## Background

Heterozygous familial hypercholesterolemia (HeFH) is one of the most common inherited metabolic disorders in humans, with an estimated prevalence of 1:311 worldwide and 1:500 to 1:200 in China in the general population [1–3]. HeFH is primarily caused by autosomal dominant variants in gene encoding low density lipoprotein receptor (*LDLR*), apolipoprotein B (*APOB*) or proprotein convertase subtilisin/kexin type 9 (*PCSK9*), leading to life-long marked elevation in circulating low density lipoprotein cholesterol (LDL-C) levels and increased risk of premature atherosclerotic coronary artery diseases [2].

Clinical practice guidelines recommend the initiation of lipid-lowering therapy as soon as possible after the diagnosis of HeFH to reduce LDL-C levels and future risk of cardiovascular diseases. Unfortunately, HeFH is largely underdiagnosed and undertreated worldwide [4, 5]. In China, with the expert consensus on familial hypercholesterolemia (FH) diagnostic criteria formulated in 2018 and the lack of widespread FH awareness, many patients were misdiagnosed or undertreated [6], with few patients on lipid-lowering medications achieving desired LDL-C levels [7]. For patients whose treatment goals cannot be achieved with maximum tolerated statins plus ezetimibe, clinical practice guidelines recommend the use of PCSK9 inhibitors. Evolocumab and alirocumab, two PCSK9 monoclonal antibodies approved for the treatment of HeFH in China, demonstrated short-term and long-term reductions in LDL-C levels in both Western and Asian populations, together with positive cardiovascular outcomes [8–11]. Despite the clear clinical evidence and benefit of PCSK9 inhibitors, their clinical use is limited by the high cost of treatment, necessitating the development of more drugs of this class to improve accessibility [12–14].

Tafolecimab is a fully human IgG2 PCSK9 monoclonal antibody developed in China. In the phase 1a study in Chinese healthy volunteers, a single dose of tafolecimab reduced LDL-C levels up to 72%. In the phase 1b study in Chinese patients with hypercholesterolemia, tafolecimab reduced LDL-C levels up to 71.6% and by more than 50% at week 12 for all tafolecimab dose regimens. In both studies, tafolecimab was well tolerated and showed an overall favorable safety profile similar to other PCSK9 antibodies [15]. We conducted the first randomized, placebo-controlled phase 3 clinical trial, CREDIT-2 (**C**linical **Re**search of **D**eveloping PCSK9 **I**nhibitor as Cholesterol-lowering **T**herapy in Chinese Patients with Dyslipidemia-**2**), to evaluate the efficacy and safety of tafolecimab in Chinese patients with HeFH.

## Methods

### Study design

This was a randomized, double-blind, placebo-controlled phase 3 study conducted from December 2019 to June 2021 across 22 study centers in China. The study protocol was approved by each center’s institutional review board or independent ethics committee. This study was conducted in accordance with the principles of Declaration of Helsinki and the International Conference on Harmonization Good Clinical Practice guidelines. All participants provided written informed consent prior to screening. The results of the study were reported in adherence to the CONSORT reporting guidelines.

### Participants

Patients (aged 18–80 years) diagnosed with definite or possible HeFH by Simon Broome criteria [16] with a screening fasting LDL-C level of ≥1.8 mmol/L (with a history of arteriosclerotic cardiovascular diseases) or ≥2.6 mmol/L (without a history of arteriosclerotic cardiovascular diseases) and on a stable lipid-lowering therapy (moderate or high intensity statins except for statin intolerance, with or without ezetimibe, niacin, omega-fatty acids) for at least 4 weeks before randomization were eligible. The full inclusion and exclusion criteria are listed in Additional file 1. During the study, participants should remain on the stable dose of moderate or high intensity statins (except for those intolerant for statins) with or without ezetimibe.

### Procedures

Eligible participants were randomized in a 2:2:1:1 ratio to receive subcutaneous tafolecimab 150 mg Q2W, tafolecimab 450 mg Q4W, placebo Q2W or placebo Q4W, respectively, in the 12-week double-blind treatment period. Randomization was implemented by an interactive web response system and was stratified by LDL-C levels at screening (≥ or < 4.8 mmol/L), by baseline ezetimibe use (yes/no) and by prior use of PCSK9 inhibitors (yes/no). The participants, investigators and study site personnel involved in treating and assessing participants were masked to treatment allocations.

After the 12-week double-blind treatment period, participants receiving tafolecimab continued to receive open-label tafolecimab with the previous regimens while participants receiving placebo Q2W or Q4W crossed over to receive open-label tafolecimab 150 mg Q2W or 450 mg Q4W, respectively, for 12 weeks, followed by an 8-week safety follow-up.

Fasting LDL-C (OSR6183, Beckman Coulter), HDL cholesterol (HDL-C, OSR6187, Beckman Coulter), total cholesterol (OSR6116, Beckman Coulter) and triglycerides (OSR61118, Beckman Coulter) concentrations were measured by commercial kits on a Beckman Coulter AU600 Chemistry Analyzer. Non-HDL cholesterol (non-HDL-C) concentration was calculated by subtracting HDL-C concentration from total cholesterol concentration. Very low density lipoprotein cholesterol (vLDL-C) concentration was calculated by dividing the triglyceride concentration by 5 (when triglyceride < 4.52 mmol/L) or by subtracting HDL-C and LDL-C concentration from total cholesterol concentration (when triglyceride ≥4.52 mmol/L). Apolipoprotein A1, apolipoprotein B and lipoprotein(a) concentrations were determined using a nephelometric method (OUED, OSAN and OQHL, respectively, Siemens BN ProSpec System). We measured unbound PCSK9 concentrations using an in-house developed enzyme-linked immunosorbent assay. All lipids and PCSK9 were tested in a central laboratory (WuXi AppTec, *Shanghai*).

DNA extracted from blood samples of all enrolled participants were sequenced by Novogene (*Beijing*) for variants in all exons of *LDLR*, *APOB*, *PCSK9* and *LDLRAP1* gene. Pathogenicity of variants were annotated using clinical classification of the Leiden Open Variation Database (LOVD) [17].

### Endpoints

The primary endpoint was the percent change from baseline to week 12 in LDL-C levels. Secondary endpoints included proportion of participants achieving ≥50% LDL-C reductions and proportion of participants with LDL-C <1.8 mmol/L at week 12 and 24, the change from baseline to week 12 in non-HDL-C, apolipoprotein B and lipoprotein(a) levels, as well as the change from baseline to week 24 in lipid levels.

Safety endpoints were assessed by adverse events, vital signs, electrocardiogram, and laboratory measurements. Adverse events were coded and classified using Medical Dictionary of Regulatory Activities (MedDRA, version 24.0). The severity and causality of adverse events were assessed by investigators based on pre-specified criteria. Immunogenicity was assessed by detecting anti-drug antibodies and neutralizing antibodies.

### Statistical analysis

Assuming a common standard deviation of 40% and a dropout rate of 10%, the sample size of 148 participants was sufficient to generate 99% power to detect ≥40% LDL-C reductions [18] in the tafolecimab 150 mg Q2W or tafolecimab 450 mg Q4W group compared with corresponding placebo group with a 0.025 two-sided significance level (therefore 0.05 for family-wise significant level).

Efficacy endpoints were analyzed in the efficacy analysis population, defined as participants who received at least one dose of tafolecimab or placebo and had at least one post-baseline assessment. Two estimands, the on-treatment estimand and the treatment policy estimand, were used to assess treatment efficacy from different perspectives and accounted for intercurrent events differently [19].

The on-treatment estimand assesses treatment efficacy prior to the intercurrent events like study drug discontinuation or background statin therapy adjustment. For continuous endpoints, mixed model for repeated measures method was used, with baseline value, baseline-by-visit interaction, visit, treatment, visit-by-treatment interaction and randomization stratification factors as fixed effects and unstructured covariance. For categorical endpoints, Clopper-Pearson method [20] was used for within-group CI calculation and Mantel-Haenszel method was used for between-group CI calculation and statistical testing, and participants without any available assessment at week 12 were imputed using last observation carried forward method.

The treatment policy estimand assesses treatment efficacy regardless of any intercurrent event. For continuous endpoints, pattern-mixture model method was used for missing data imputation and analysis of covariance (ANCOVA) was used for analysis of imputed data. For categorical endpoints, statistical testing methods were the same as those of the on-treatment estimand except that participants without any available assessment at week 12 were treated as non-responders.

The primary endpoint (the percent change from baseline to week 12 in LDL-C levels) and key secondary endpoints (proportion of participants achieving ≥50% LDL-C reductions and proportion of participants with LDL-C level <1.8 mmol/L at week 12) were assessed by both on-treatment and treatment policy estimands, and controlled for type I error. The percent change from baseline to week 12 in non-HDL-C, apolipoprotein B and lipoprotein (a) levels were assessed by on-treatment estimand. Other efficacy endpoints were reported descriptively.

Safety analyses were done on the safety analysis population, defined as all participants who received at least one dose of tafolecimab or placebo. Adverse events were descriptively summarized.

Exploratory analyses were done to evaluate the effects of *LDLR* variant status and pathogenicity on 12-week reductions in LDL-C levels. To this end, we used the change in LDL-C levels from baseline to week 12 for the tafolecimab groups and change in LDL-C levels from week 12 to week 24 for the placebo groups.

All statistical analyses were performed with SAS version 9.4.

## Results

### Participants

**Fig. 1:**
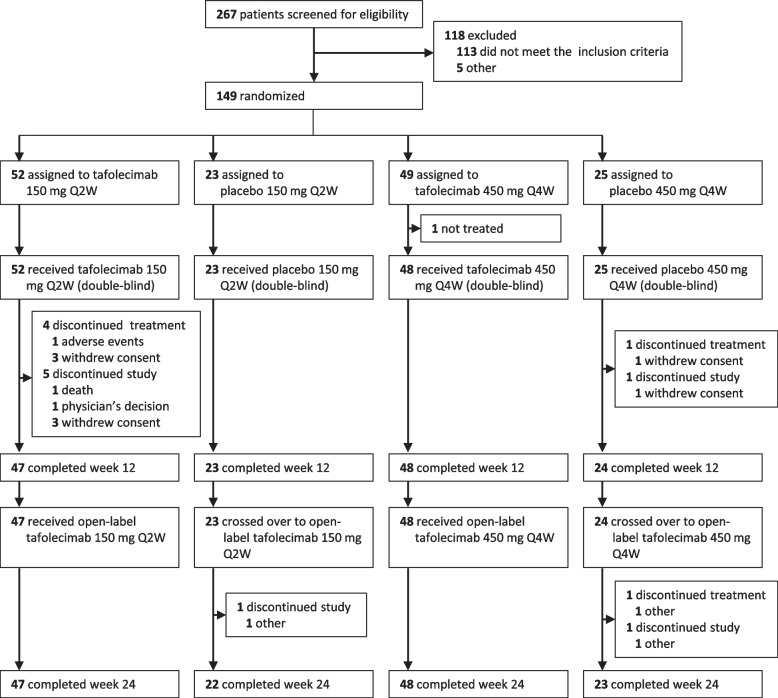
Study flow. Q2W = every 2 weeks; Q4W = every 4 weeks

Of 267 patients screened, 149 were randomized. 148 participants received at least one dose of tafolecimab (*n* = 100) or placebo (*n* = 48) in the double-blind treatment period and were included in the efficacy and safety analysis population. Four participants in the tafolecimab 150 mg Q2W group and one in the placebo groups prematurely discontinued the double-blind treatment. One hundred forty-two participants (95.9%) completed week 12 and received the open-label tafolecimab treatment. A total of 140 participants (94.6%) completed week 24 (Fig. 1). Participant demographics and baseline characteristics were generally balanced between treatment groups (Table 1).

**Table 1:** Participants demographics and baseline characteristics

	150 mg Q2W		450 mg Q4W	
	Tafolecimab (***n*** = 52)	Placebo (***n*** = 23)	Tafolecimab (***n*** = 48)	Placebo (***n*** = 25)
**Age (years)**	48.7 (13.2)	48.0 (14.4)	51.9 (10.7)	47.4 (14.2)
**Male sex, n (%)**	29 (55.8)	11 (47.8)	21 (43.8)	16 (64.0)
**Coronary artery disease**^**a**^**, n (%)**	23 (44.2)	5 (21.7)	19 (39.6)	9 (36.0)
**Screening LDL-C**^**b**^**, n (%)**				
<4.8 mmol/L	38 (73.1)	16 (69.6)	36 (75.0)	18 (72.0)
≥4.8 mmol/L	14 (26.9)	7 (30.4)	12 (25.0)	7 (28.0)
**Lipid-regulating medication, n (%)**				
High dose statin^c^	9 (17.3)	3 (13.0)	4 (8.3)	2 (8.0)
Moderate dose statin	43 (82.7)	20 (87.0)	43 (89.6)	23 (92.0)
Ezetimibe^b^	15 (28.8)	5 (21.7)	15 (31.3)	7 (28.0)
**HeFH classification**^**d**^**, n (%)**				
Definite	42 (80.8)	22 (95.7)	33 (68.8)	22 (88.0)
Possible	10 (19.2)	1 (4.3)	15 (31.3)	3 (12.0)
**Lipid parameters**				
LDL-C (mmol/L)	4.25 (1.11)	4.27 (0.98)	4.21 (1.14)	4.32 (1.22)
Non-HDL-C (mmol/L)	4.65 (1.28)	4.72 (1.10)	4.60 (1.33)	4.75 (1.37)
Apolipoprotein B (g/L)	1.20 (0.30)	1.21 (0.27)	1.19 (0.31)	1.23 (0.31)
Lipoprotein(a) (g/L)	0.29 (0.09-0.53)	0.32 (0.10-0.46)	0.19 (0.10-0.50)	0.28 (0.12-0.49)
**Unbound PCSK9 (ng/mL)**	707.28 (231.05)	795.74 (218.57)	785.03 (234.15)	708.71 (250.91)

Data are mean (SD), median (interquartile range) or n (%)

*HeFH* heterozygous familial hypercholesterolemia, *LDL-C* low density lipoprotein cholesterol, *non-HDL-C* non-high density lipoprotein cholesterol, *Q2W* every 2 weeks, *Q4W* every 4 weeks

^a^Coronary artery diseases include previous acute coronary syndrome (myocardial infarction or unstable angina), stable angina, coronary revascularization (percutaneous coronary intervention, coronary artery bypass graft surgery and other arterial revascularization procedures), and significant plaque on coronary angiography, computed tomography scan (multivessel coronary disease with two major epicardial arteries having >50% stenosis)

^b^Randomization stratification factors

^c^Defined as atorvastatin 40–80 mg or rosuvastatin 20 mg

^d^By Simon Broome diagnostic criteria

### Efficacy

**Fig. 2:**
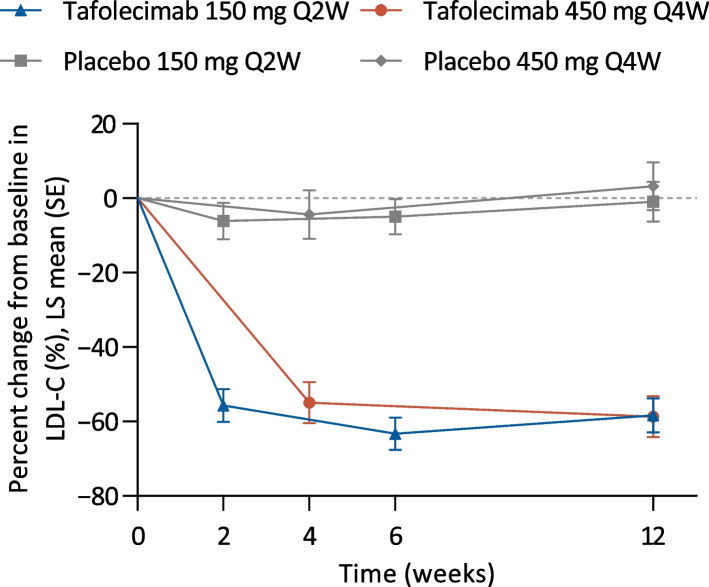
Percent change from baseline in LDL-C levels over time for each treatment group during the double-blind treatment period (on-treatment estimand). LDL-C = low density lipoprotein cholesterol; LS = least squares; Q2W = every 2 weeks; Q4W = every 4 weeks; SE = standard error

Tafolecimab treatment led to robust and persistent reductions in LDL-C levels (Fig. 2; Additional file 2: Fig. S1; Additional file 3: Table S1 and S2). For the on-treatment estimand, the percent change from baseline to week 12 in LDL-C levels were −58.4% with tafolecimab 150 mg Q2W and −1.0% with placebo Q2W (treatment difference, −57.4% [97.5% CI, −69.2 to −45.5], *P* <0.0001); −58.7% with tafolecimab 450 mg Q4W and 3.2% with placebo Q4W (treatment difference, −61.9% [−73.4 to −50.4], *P* <0.0001) (Table 2). For the treatment policy estimand, the treatment difference versus placebo were −54.6% [97.5% CI, −66.5% to −42.6%] for tafolecimab 150 mg Q2W; −60.7% [−71.3 to −50.0] for tafolecimab 450 mg Q4W (both *P* <0.0001) (Additional file 3: Table S1). Consistent reductions in LDL-C levels were observed across all subgroups at week 12 and were not mediated by sex, ezetimibe use, baseline PCSK9 levels, statin intensity or presence of coronary artery disease (Additional file 2: Fig. S2). Significantly more participants receiving tafolecimab achieved ≥50% LDL-C reductions and LDL-C<1.8 mmol/L at week 12 than those receiving placebo (both estimands, *P* <0.0001 for all comparison versus placebo) (Table 2; Additional file 2: Fig. S2). Moreover, in the very-high-risk population (*n* = 125), assessed by on-treatment estimand at week 12, LDL-C <1.4 mmol/L was achieved in 15 participants (34.9%) receiving tafolecimab 150 mg Q2W (*n* = 43) and 19 (44.2%) receiving tafolecimab 450 mg Q4W (*n* = 43), as compared with one receiving placebo (*n* = 39). Reductions in LDL-C levels and LDL-C target attainment rates were essentially maintained through week 24 with both tafolecimab regimens (Additional file 3: Table S2).

**Table 2:** Main efficacy endpoints at week 12 assessed by on-treatment estimand

	150 mg Q2W			450 mg Q4W		
	Tafolecimab (***n*** = 52)	Placebo (***n*** = 23)	ETD versus placebo	Tafolecimab (***n*** = 48)	Placebo (***n*** = 25)	ETD versus placebo
**LDL-C**						
Percent CFB (%)^a^	−58.4 (4.6)	−1.0 (5.3)	−57.4 (−69.2, −45.5); *P* <0.0001	−58.7 (5.5)	3.2 (6.4)	−61.9 (−73.4, −50.4) ; *P* <0.0001
CFB (mmol/L)	−2.42 (0.22)	0.05 (0.25)	−2.47 (−2.96, −1.99) ; *P* <0.0001	−2.38 (0.25)	0.10 (0.29)	−2.48 (−2.93, −2.03) ; *P* <0.0001
≥50% reduction^a^	31 (59.6)	0	58.5 (42.2, 74.8) ; *P* <0.0001	36 (75.0)	0	73.4 (58.5, 88.4) ; *P* <0.0001
<1.8 mmol/L^a^	27 (51.9)	1 (4.3)	47.9 (29.2, 66.7) ; *P* <0.0001	30 (62.5)	0	59.9 (43.2, 76.5) ; *P* <0.0001
**Non-HDL-C**						
Percent CFB (%)	−60.6 (4.9)	−2.8 (5.6)	−57.8 (−68.6, −47.0) ; *P* <0.0001	−60.3 (5.5)	0.4 (6.5)	−60.7 (−70.7, −50.6) ; *P* <0.0001
CFB (mmol/L)	−2.74 (0.25)	0.01 (0.29)	−2.75 (−3.30, −2.20) ; *P* <0.0001	−2.61 (0.29)	0.04 (0.34)	−2.65 (−3.17, −2.12) ; *P* <0.0001
**Apolipoprotein B**						
Percent CFB (%)	−56.7 (6.2)	5.0 (8.0)	−61.7 (−79.1, −44.3) ; *P* <0.0001	−56.8 (4.9)	−1.0 (5.8)	−55.8 (−64.5, −47.0) ; *P* <0.0001
CFB (g/L)	−0.66 (0.08)	0.07 (0.09)	−0.74 (−0.94, −0.54) ; *P* <0.0001	−0.65 (0.07)	−0.02 (0.08)	−0.63 (−0.75, −0.51) ; *P* <0.0001
**Lipoprotein(a)**						
Percent CFB (%)	−47.6 (7.2)	−4.3 (8.2)	−43.3 (−58.9, −27.6) ; *P* <0.0001	−30.0 (7.1)	4.1 (8.2)	−34.1 (−47.1, −21.1) ; *P* <0.0001
CFB (g/L)	−0.17 (0.02)	−0.04 (0.03)	−0.13 (−0.18, −0.08) ; *P* <0.0001	−0.13 (0.02)	−0.01 (0.03)	−0.12 (−0.17, −0.08) ; *P* <0.0001
≥50% reduction	16 (30.8)	1 (4.3)	27.6 (11.2, 43.9) ; *P* =0.0010	20 (41.7)	0	42.2 (27.8, 56.5) ; *P* <0.0001

Data are least squares mean (standard error) for CFB and percent CFB; n (%) for LDL-C and lipoprotein(a) target attainment rates; least squares mean (97.5% CI) for ETD of percent CFB in LDL-C; mean (97.5% CI) for ETD of LDL-C target attainment rates; least squares mean (95% CI) for ETD of CFB in LDL-C, as well as ETD of CFB and percent CFB in other lipid parameters; mean (95% CI) for ETD of lipoprotein(a) target attainment rates.

*CFB* change from baseline, *CI* confidence interval, *ETD* estimated treatment difference, *LDL-C* low density lipoprotein cholesterol, *non-HDL-C* non-high density lipoprotein cholesterol, *Q2W* every 2 weeks, *Q4W* every 4 weeks

^a^Controlled for type I error (pairwise α = 0.025).

Tafolecimab treatment markedly reduced lipoprotein(a) levels (Table 2; Additional file 3: Table S2). The percent change from baseline to week 12 in lipoprotein(a) levels assessed by on-treatment estimand was −47.6% with tafolecimab 150 mg Q2W and −4.3% with placebo Q2W (treatment difference, −43.3% [95% CI, −58.9 to −27.6], *P* <0.0001); −30.0% with tafolecimab 450 mg Q4W and 4.1% with placebo Q4W (treatment difference, −34.1% [−47.1 to −21.1], *P* <0.0001) (Table 2). Significantly more participants receiving tafolecimab achieved ≥50% reductions in lipoprotein(a) level at week 12 than those receiving placebo (on-treatment estimand) (Table 2). In an exploratory subgroup analysis in participants with baseline lipoprotein(a) levels ≥50 mg/dl (*n* = 40), the percent change from baseline in lipoprotein(a) levels at week 12 were −44.4% with tafolecimab 150 mg Q2W and −8.7% with placebo Q2W (treatment difference, −35.7% [95% CI, −59.1 to −12.4], *P* =0.0058); −48.8% with tafolecimab 450 mg Q4W and −16.5% with placebo Q4W (treatment difference, −32.3% [−52.6 to −12.0], *P* =0.0041).

In addition, tafolecimab treatment also led to significant and persistent reductions in non-HDL-C and apolipoprotein B levels (Table 2; Additional file 3: Table S2). Greater benefits including reductions in total cholesterol, vLDL-C and triglycerides levels, and increase in HDL-C levels were observed in participants receiving tafolecimab as compared with placebo (Additional file 3: Table S2). In alignment with the overall improvement in lipids, unbound PCSK9 levels were markedly suppressed throughout the study in participants receiving tafolecimab (Additional file 3: Table S2).

Of 148 participants, 102 had *LDLR* variants; 18 had *APOB* variants; 1 had *PCSK9* variant; 2 had *LDLRAP1* variants; and 38 had no variant in exons of these four genes. Among 102 participants harboring *LDLR* variants, 37 (36.3%) had single nonsense variants, splicing junction variants or frameshift variants that led to putative premature translation termination, collectively classified as pathogenic *LDLR* variants; 58 (56.9%) had single likely pathogenic *LDLR* variants. Two variants were identified in 13 double heterozygotes (11 with both *LDLR* and *APOB* variants and 2 with both *LDLR* and *LDLRAP1* variants) and 7 compound heterozygotes (with two *LDLR* variants).

Participants with compound heterozygous *LDLR* variants tended to have higher baseline lipid and PCSK9 levels and have poor lipid-lowering responses to tafolecimab treatment (Additional file 2: Fig. S3 and S4). *APOB*, *LDLRAP1* or *PCSK9* variants identified in this study did not affect the lipid-lowering efficacy of tafolecimab (data not shown). With given *LDLR* variant pathogenicity, reductions from baseline to week 12 in LDL-C levels were similar with different dose regimens (Additional file 2: Fig. S3). Reductions in lipids and PCSK9 levels were generally comparable among participants with single *LDLR* variants and those without (Additional file 2: Fig. S4).

### Safety

Tafolecimab was well tolerated and showed an overall favorable safety profile (Table 3). One participant receiving tafolecimab 150 mg Q2W reported dermatitis allergic, moderate in severity and leading to study drug discontinuation during the double-blind treatment period. Serious adverse events were reported in two participants receiving tafolecimab 150 mg Q2W and two receiving tafolecimab 450 mg Q4W during the double-blind treatment period, all unrelated to the study drug according to the investigators’ judgement. One participant in the tafolecimab 150 mg Q2W group with a history of hypertension and coronary artery disease and long-term use of aspirin experienced a serious adverse event of upper gastrointestinal hemorrhage after six doses of tafolecimab. The event led to study drug discontinuation and subsequent death (a narrative provided in Additional file 4). Another participant in the same group reported a serious adverse event of angina unstable. Two participants receiving tafolecimab 450 mg Q4W reported serious adverse events, one with angina unstable and one with coronary artery disease.

**Table 3:** Treatment-emergent adverse events and laboratory abnormalities in the double-blind treatment period

	150 mg Q2W		450 mg Q4W	
	Tafolecimab (***n*** = 52)	Placebo (***n*** = 23)	Tafolecimab (***n*** = 48)	Placebo (***n*** = 25)
**Adverse events**				
Any	36 (69.2)	13 (56.5)	23 (47.9)	15 (60.0)
Serious	2 (3.8)	0	2 (4.2)	0
Deaths	1 (1.9)	0	0	0
Leading to treatment discontinuation	2 (3.8)	0	0	0
**Most common adverse events in participants receiving tafolecimab**^**a**^				
Upper respiratory tract infection	11 (21.2)	1 (4.3)	2 (4.2)	3 (12.0)
Blood creatine phosphokinase increased	3 (5.8)	0	3 (6.3)	1 (4.0)
Alanine aminotransferase increased	4 (7.7)	0	0	0
Aspartate aminotransferase increased	4 (7.7)	0	0	0
Hypertension	4 (7.7)	0	0	0
**Muscle-related adverse events**^**b**^	5 (9.6)	1 (4.3)	0	2 (8.0)
**Hypersensitivity**^**c**^	5 (9.6)	1 (4.3)	0	2 (8.0)
**Potential injection-site adverse events**^**d**^	2 (3.8)	0	1 (2.1)	0
**Laboratory results**				
ALT or AST >3× ULN (any post-baseline value)	1 (1.9)	0	1 (2.1)	0
Creatine kinase >5× ULN (any post-baseline value)	0	1 (4.3)	2 (4.2)	0

Data are number of participants (%)

*ALT* alanine aminotransferase, *AST* aspartate aminotransferase, *MedDRA* The Medical Dictionary for Regulatory Activities, *Q2W* every 2 weeks, *Q4W* every 4 weeks, *ULN* upper limit of normal

^a^By MedDRA (version 24.0) preferred term

^b^Defined using statin-associated muscle events (ACC/AHA guideline on the management of blood cholesterol [2018])

^c^Defined as acute systemic reactions characterized by pruritus, generalized flush, urticaria, angioedema, respiratory distress or hypotension

^d^Defined as events characterized by injection site swelling, injection site erythema, injection site haemorrhage, injection site pruritus, injection site induration or injection site pain

During the double-blind treatment period, treatment-emergent adverse events (TEAEs) were reported in 59% of participants receiving tafolecimab and in 58% receiving placebo. Except for the serious adverse events of upper gastrointestinal hemorrhage and angina unstable reported in the tafolecimab 450 mg Q4W group, all TEAEs reported during the double-blind treatment period were mild or moderate in severity. The most commonly-reported TEAEs in the tafolecimab groups included upper respiratory tract infection, increased blood creatine phosphokinase, increased alanine aminotransferase, increased aspartate aminotransferase and hypertension (Table 3). Rates of muscle-related adverse events and hypersensitivity were similar between the tafolecimab and placebo groups (5.0% with tafolecimab vs 6.3% with placebo). Injection-site adverse events were observed but infrequent in the tafolecimab groups (3.0%), all mild in severity.

During the double-blind treatment period, one participant receiving tafolecimab 450 mg Q4W with baseline alanine aminotransferase level over two times the upper limit of normal (ULN) had ALT elevation over three times ULN. One participant receiving tafolecimab 150 mg Q2W with baseline aspartate aminotransferase level slightly exceeding ULN had AST elevation over three times ULN (Table 3). All other ALT and AST elevation were within three times ULN. Adverse events associated with ALT or AST abnormality included alanine aminotransferase increased, aspartate aminotransferase increased and hepatic function abnormal, all mild in severity.

One participant in the tafolecimab 150 mg group developed anti-drug antibody during the double-blind treatment period and neutralizing antibody remained negative throughout the study.

The safety profile of tafolecimab in the open-label treatment period was generally similar to the double-blind treatment period, with no additional safety issues identified (Additional file 3: Table S3).

## Discussion

HeFH is significantly underdiagnosed and undertreated in many countries and the situation is even worse in China, posing a great threat to the patients and necessitating earlier and more effective pharmacological intervention on the basis of statins and ezetimibe [6, 12]. To the best of our knowledge, this study was the first large-scale randomized, placebo-controlled clinical trial in Chinese patients with HeFH. By demonstrating the robust efficacy and overall favorable safety of tafolecimab, this study added important evidence of the applicability of PCSK9 antibodies in Chinese patients with HeFH while picturing a comprehensive genetic profile and clinical feature of the disease population. Moreover, with the upcoming marketing of tafolecimab as the first domestic PCSK9 monoclonal antibody in China, tafolecimab is expected to increase the affordability and accessibility to PCSK9 inhibitors in Chinese patients with FH and satisfy the unmet medical needs thus improving their clinical outcomes.

The average age of HeFH diagnosis in this study (49.6 years) was consistent with those reported for East Asian or Chinese patients with HeFH [7, 12, 21]. Many participants were previously misdiagnosed and treated as dyslipidemia and the HeFH disease was diagnosed on the study entry, further reflecting that HeFH is significantly underdiagnosed in China. Most participants (87.2%) were on stable statins of moderate intensity, which is in consistency with the fact that high-intensity statins are not well tolerated and seldom prescribed in the clinics in China [22]. The large gap of the baseline LDL-C level (mean 4.26 mmol/L) from the guideline-recommended target level underlies the necessity of combination with additional lipid-lowering therapies [7, 12].

In Chinese HeFH participants with higher baseline LDL-C levels and higher proportion of moderate-intensity statin use, the LDL-C-lowering effect of tafolecimab was generally similar to those of other approved PCSK9 antibodies observed in western population. In RUTHERFORD-2 trial, evolocumab 140 mg Q2W and 420 mg monthly achieved placebo-adjusted LDL-C reductions of 59.2% and 61.3% at week 12, respectively, in patients with HeFH [9]. In ODYSSEY FH trials, alirocumab dosed up to 150 mg Q2W achieved placebo-adjusted LDL-C reductions ranging from 51.4% to 57.9% at week 24 [8]. In our study, placebo-adjusted mean reductions in LDL-C levels at week 12 were 57.4% with tafolecimab 150 mg Q2W and 61.9% with tafolecimab 450 mg Q4W. In all these studies, the proportion of patients achieving LDL-C <1.8 mmol/L were significantly higher in patients receiving PCSK9 inhibitors than those on statin or ezetimibe alone. Indeed, according to the European Atherosclerosis Society (EAS) Familial Hypercholesterolemia Studies Collaboration (FHSC) global registry, use of PCSK9 inhibitors among patients with HeFH was associated with greater odds of having LDL-C lower than 1.8 mmol/L [12]. However, only 2.4% of the patients were taking PCSK9 inhibitors in combination with statin or ezetimibe [12]. Our study provided the first evidence that robust and persistent reductions in LDL-C levels can be achieved with PCSK9 antibodies in Chinese patients with HeFH, demonstrating a potential new option for the great unmet clinical needs.

Another benefit of PCSK9 inhibition is reductions in lipoprotein(a) levels, a LDL-like lipoprotein associated with cardiovascular diseases. Cardiovascular outcome trials of alirocumab and evolocumab both suggested that lipoprotein(a) level lowering with PCSK9 inhibition was associated with a greater cardiovascular risk reduction beyond LDL-C lowering alone, indicating additional benefits from further reduction of lipoprotein(a). Current PCSK9 antibodies lowered lipoprotein(a) levels by approximately 25%, but were least effective in patients with high lipoprotein(a) level (>50 mg/dl), with a 14% reduction [23]. In this study, 34.1–43.3% placebo-adjusted reductions in lipoprotein(a) levels were achieved in overall population and 32.3–35.7% placebo-adjusted reductions were achieved in participants with baseline lipoprotein(a) levels ≥50 mg/dl. Of note, given the lower lipoprotein(a) levels in Chinese population [8] and skewed distribution of lipoprotein(a) levels, together with the suboptimal mass units used in this study, the comparison of lipoprotein(a)-lowering effect with other PCSK9 inhibitors is premature and the potential of tafolecimab warrants further investigation.

Consistent with previously reported genetic profiles of HeFH, potential causative variants were most commonly detected in *LDLR* gene. By exploratory analysis aggregating 12-week efficacy from the tafolecimab groups in the double-blind treatment period and the placebo groups in the open label period, we observed that participants with single variants in *LDLR* responded equally well to those without *LDLR* variants. This result, in accordance with data from other PCSK9 inhibitors, indicates that this medication class can effectively reduce lipids levels in patients with only one copy of *LDLR* full functional [9, 24, 25].

Seven compound heterozygotes with two *LDLR* variants were enrolled in this study. Among five participants receiving tafolecimab in the double-blind treatment period, four had markedly compromised reductions in LDL-C levels (Additional file 2: Fig. S3). Of note, most *LDLR* variants identified in these compound heterozygotes were missense variants and annotated as likely pathogenic according to LOVD. One participant with two likely pathogenic *LDLR* variants had a 49.3% LDL-C reduction from baseline at week 12. Obviously, functional annotations of *LDLR* variants, especially missense variants, based on which effects of PCSK9 inhibitors were established, remain a significant challenge.

In this study, with 84.5% of participants at very-high cardiovascular risk, tafolecimab demonstrated a favorable risk-benefit profile, with safety profile similar to those identified in previous studies and those of alirocumab and evolocumab [8, 9, 11, 15, 26]. Serious adverse events were possibly related to underlying medical conditions of the participants, further reflecting late diagnosis of HeFH in China and high risk for cardiovascular diseases.

A major limitation of this study was the short treatment duration. Although improvements on lipid profiles were evident after 24-week treatment with tafolecimab, a future study is warranted to evaluate its long-term efficacy and cardiovascular benefits in patients with HeFH. Another limitation was that all participants were Chinese, limiting the generalizability of the results to patients of other races or ethnicities.

## Conclusions

In summary, tafolecimab dosed 150 mg Q2W and 450 mg Q4W demonstrated robust and persistent lipid-lowering efficacy in Chinese patients with HeFH. Participants on tafolecimab achieved a 12-week reduction in LDL-C levels around 60% and were significantly more likely to attain ≥50% LDL-C reductions and LDL-C<1.8 mmol/L. Together with the favorable safety profile, tafolecimab may offer HeFH patients a novel treatment option for effective disease control.

## Supplementary Information

**Additional file 1.** Full inclusion and exclusion criteria.

**Additional file 2: Fig. S1**. LDL-C levels at baseline and week 12 for each participant with both baseline and week 12 LDL-C measurements. **Fig. S2**. Subgroup analysis on percent change from baseline to week 12 in LDL-C levels. **Fig. S3**. Percent change from baseline at week 12 in LDL-C levels for each tafolecimab-treated participant by *LDLR* variant pathogenicity. **Fig. S4**. Baseline and 12-week change in lipids and PCSK9 levels by *LDLR* variant pathogenicity.

**Additional file 3: Table S1.** Efficacy endpoints at week 12 assessed by treatment policy estimand. **Table S2.** Descriptive statistics for endpoints in lipids and unbound PCSK9 levels at week 12 and week 24. **Table S3.** Most commonly-reported treatment-emergent adverse events (≥5% in any group) in participants receiving tafolecimab during the 12-week double-blind treatment period and 24-week treatment period.

**Additional file 4.** Narrative for the death.

## Data Availability

The data supporting the analyses contained in the manuscript will be made available upon reasonable written request from researchers whose proposed use of the data for a specific purpose has been approved by the corresponding authors.

## Abbreviations

AE: Adverse event
ANCOVA: Analysis of covariance
FH: Familial hypercholesterolemia
HeFH: Heterozygous familial hypercholesterolemia
LDL-C: Low density lipoprotein cholesterol
non-HDL-C: Non-high density lipoprotein cholesterol
PCSK9: Proprotein convertase subtilisin/kexin type 9
Q2W: Every 2 weeks
Q4W: Every 4 weeks
TEAE: Treatment-emergent adverse event
ULN: Upper limit of normal
vLDL-C: Very low density lipoprotein cholesterol
