# Supplementary material for: Efficacy and safety of tafolecimab in Chinese patients with heterozygous familial hypercholesterolemia: a randomized, double-blind, placebo-controlled phase 3 trial (CREDIT-2)
Source: BMC Med. 2023 Feb 28;21:77. doi: 10.1186/s12916-023-02797-8 (PMC9976471; doi:10.1186/s12916-023-02797-8)
Supplement: Supplementary file 1 — Additional file 1. Full inclusion and exclusion criteria. [file 12916_2023_2797_MOESM1_ESM.docx]

**Full inclusion and exclusion criteria**

| **Inclusion criteria** |
| --- |
| 1. Provide signed and dated informed consent.  2. Male or female ≥ 18 and ≤ 80 years of age at the time of screening.  3. Body weight ≥ 40 kg at screening.  4. Definite or possible HeFH according to the UK Simon Broome (SBR) criteria:  Definite HeFH: i.e., total cholesterol > 7.5 mmol/L, or LDL-C concentration > 4.9 mmol/L, and meet at least one of the following: 1) presence of tendon xanthoma in the patient or his/her relative (at least one of the first or second degree relatives); 2) evidence of gene mutations in LDL receptor, ApoB-100, or PCSK9;  Possible HeFH: i.e., total cholesterol > 7.5 mmol/L, or LDL-C concentration > 4.9 mmol/L and meet at least one of the following: 1) history of myocardial infarction in a second-degree relative before the age of 50 years or in a first-degree relative before the age of 60 years; 2) history of total cholesterol > 7.5 mmol/L in a first-degree or second-degree adult relative or history of total cholesterol > 6.7 mmol/L in a child, brother, or sister 16 years of age or before the age of 16 years.  5. Maintain a low-fat diet and is on a stable lipid-lowering therapy (moderate-intensity and above statins, except for statin intolerance, with or without ezetimibe, niacin, omega-fatty acids) for at least 4 weeks. If on fibrates, stable treatment with fibrates for at least 6 weeks.  6. Fasting LDL cholesterol concentration ≥ 1.8 mmol/L in patients with a history of atherosclerotic cardiovascular disease at screening; Fasting LDL cholesterol concentration ≥ 2.6 mmol/L in patients without a history of atherosclerotic cardiovascular disease.  7. Patient indicates willingness and cooperation to complete all procedures and study intervention cycles in the study. |
| **Exclusion criteria** |
| 1. Patients with a diagnosis of homozygous familial hypercholesterolemia.  2. Prior dialysis or plasmapheresis within 4 months prior to screening.  3. Patients with previous liver transplantation.  4. Patient had a change in regimen or dose of statins, ezetimibe, niacin, and omega-fatty acids within 4 weeks prior to randomization (these patients may be rescreened after 1 month of stable lipid-lowering medication).  5. New York Heart Association (NYHA) Class III or IV heart failure, or recent left ventricular ejection fraction ≤ 30%.  6. Poorly controlled serious cardiac arrhythmia, defined as recurrent and highly symptomatic ventricular tachycardia, atrial fibrillation with rapid ventricular rate, or supraventricular tachycardia.  7. Previous myocardial infarction, unstable angina, percutaneous coronary intervention, coronary artery bypass grafting within 3 months or previous stroke within 3 months prior to enrollment.  8. Planned percutaneous coronary intervention, coronary artery bypass grafting, or other cardiac surgery during the study.  9. Type 1 diabetes mellitus or poorly controlled blood glucose (HbA1c > 8.5%), or type 2 diabetes mellitus requiring multiple daily insulin injections.  10. Presence of uncontrolled clinical conditions that may affect lipid or lipoprotein levels (patients on thyroid hormone replacement therapy, whose thyroid hormone dose needs to be stable for at least 6 weeks prior to the screening visit). Poorly controlled hypothyroidism or hyperthyroidism, defined as TSH < lower limit of normal, or > 1.5 times upper limit of normal.  11. Poorly controlled hypertension, defined as sitting systolic blood pressure > 180 mmHg or diastolic blood pressure > 110 mmHg, confirmed by repeated measurements.  12. Severe renal impairment, defined as estimated glomerular filtration rate < 30 mL/min/1.73m^2^ at screening.  13. Active liver disease or impaired liver function, defined as aspartate aminotransferase or alanine aminotransferase > 3 x ULN as determined by local laboratory analysis at screening.  14. Creatine kinase (CK) ≥ 3 x ULN at screening.  15. Presence of known active infection or major hematologic, renal, metabolic, gastrointestinal or endocrine dysfunction, as judged by the investigator.  16. Previous diagnosis of deep vein thrombosis or pulmonary embolism.  17. Female patients with potential to become pregnant, except those who have been sterilized or have been amenorrheic, are unwilling to inform their sexual partners of their participation in the clinical study and to take effective contraception measures during the administration of the study drug and for 15 weeks after the last dose of the study drug. Male patients who are unwilling to inform their female partners of their participation in this clinical study.  18. Patients who are pregnant or lactating, or who plan to become pregnant or lactating while taking study drug or within 15 weeks after the last dose of study drug.  19. Has a malignancy within the past 5 years (except non-melanoma skin cancer, carcinoma in situ of the cervix, ductal carcinoma in situ of the breast, or stage 1 prostate cancer).  20. Patient has received treatment with a PCSK9 inhibitor or has participated in another study that inhibits PCSK9 within 4 months prior to randomization.  21. Known hypersensitivity to the study medication and its components.  22. Unsuitable for participation in the study as judged by the investigator (e.g., alcohol or other drug abuse, inability or unwillingness to comply with the protocol, or psychiatric illness).  23. Currently participating in another medical device or drug study, or the end of a previous medical device or drug clinical study, or receiving another investigational drug less than 30 days ago.  24. Presence of factors that, in the opinion of the investigator, may have an impact on the safety of the patient.  25. Positive for HIV (HIV) antibody, hepatitis C (HCV) antibody, or syphilis antibody at screening.  26. Hepatitis B surface antigen (HBsAg) and/or hepatitis B core antibody (HBcAb) positive and hepatitis B virus DNA copies ≥ 1000 IU/ml. |
