# Supplementary material for: Efficacy and safety of tafolecimab in Chinese patients with heterozygous familial hypercholesterolemia: a randomized, double-blind, placebo-controlled phase 3 trial (CREDIT-2)
Source: BMC Med. 2023 Feb 28;21:77. doi: 10.1186/s12916-023-02797-8 (PMC9976471; doi:10.1186/s12916-023-02797-8)
Supplement: Supplementary file 2 — Additional file 2: Fig. S1. LDL-C levels at baseline and week 12 for each participant with both baseline and week 12 LDL-C measurements. Fig. S2. Subgroup analysis on percent change from baseline to week 12 in LDL-C levels. Fig. S3. Percent change from baseline at week 12 in LDL-C levels for each tafolecimab-treated participant by LDLR variant pathogenicity. Fig. S4. Baseline and 12-week change in lipids and PCSK9 levels by LDLR variant pathogenicity. [file 12916_2023_2797_MOESM2_ESM.pdf]

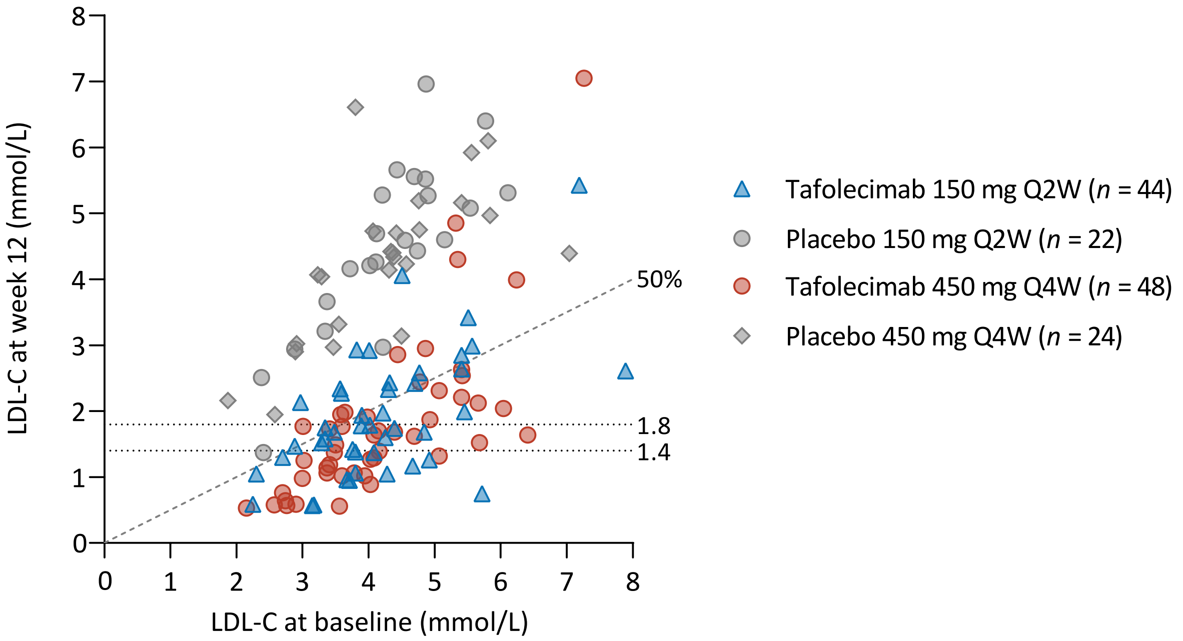

Fig. S1. LDL-C levels at baseline and week 12 for each participant with both baseline and week 12 LDL-C measurements. The dashed line indicates the boundary for 50% reduction from baseline to week 12 in LDL-C levels. LDL-C = low density lipoprotein cholesterol; Q2W = every 2 weeks; Q4W = every 4 weeks.

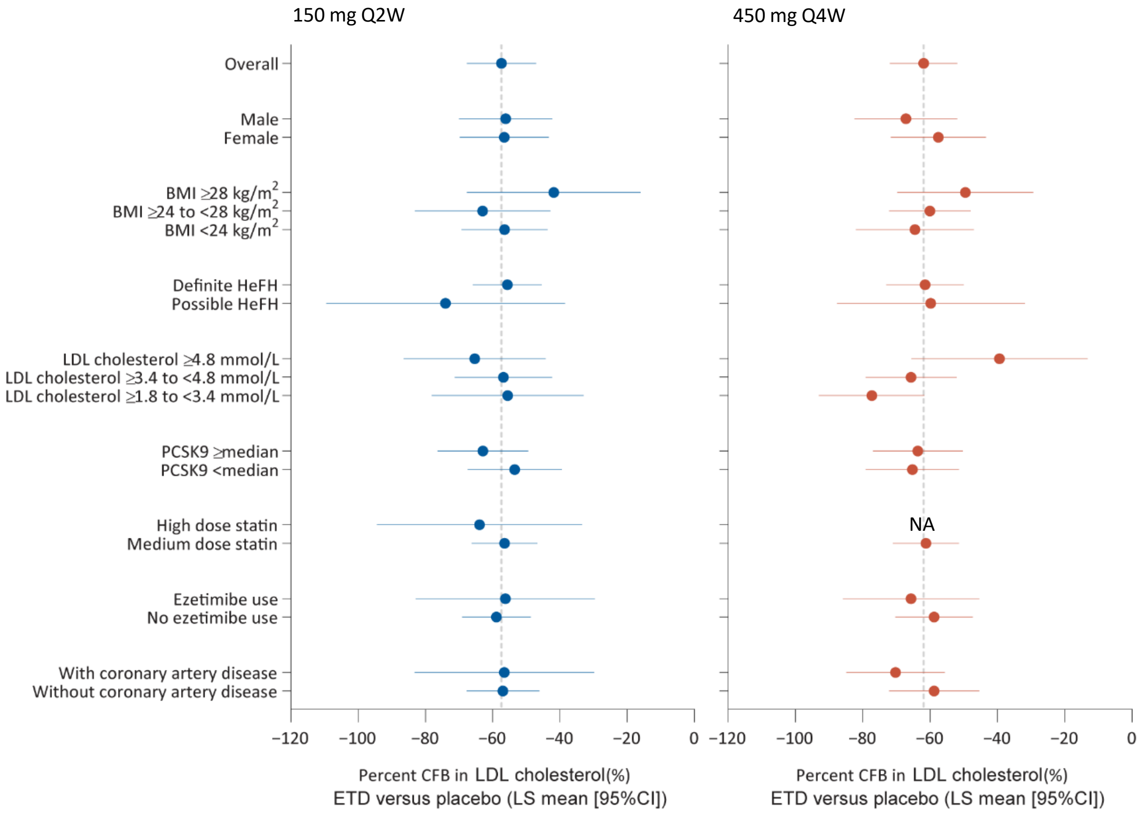

Fig. S2. Subgroup analysis on percent change from baseline to week 12 in LDL-C levels. BMI = body mass index; CFB = change from baseline; CI = confidence interval; ETD = estimated treatment difference; HeFH = heterozygous familial hypercholesterolemia; LDL-C = low density lipoprotein cholesterol; LS = least squares; NA = not available; Q2W = every 2 weeks; Q4W = every 4 weeks.

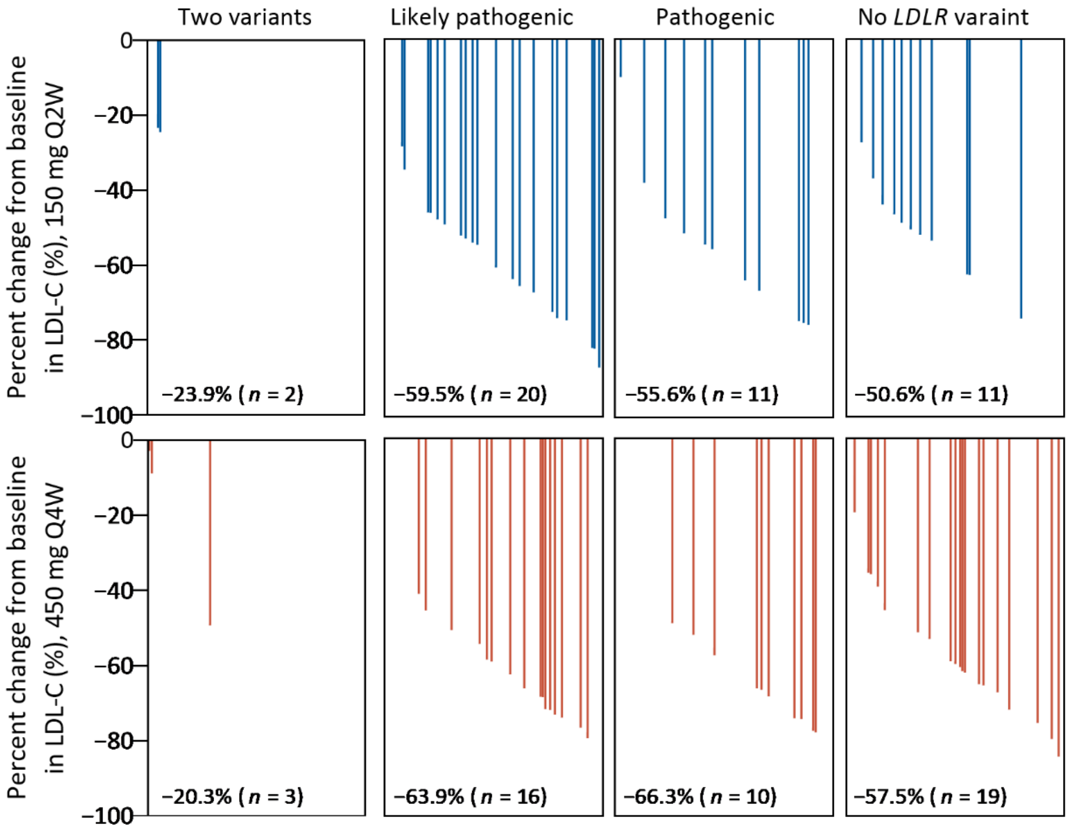

Fig. S3. Percent change from baseline at week 12 in LDL-C levels for each tafolecimab-treated participant by *LDLR* variant pathogenicity. LDL-C = low density lipoprotein cholesterol; Q2W = every 2 weeks; Q4W = every 4 weeks.

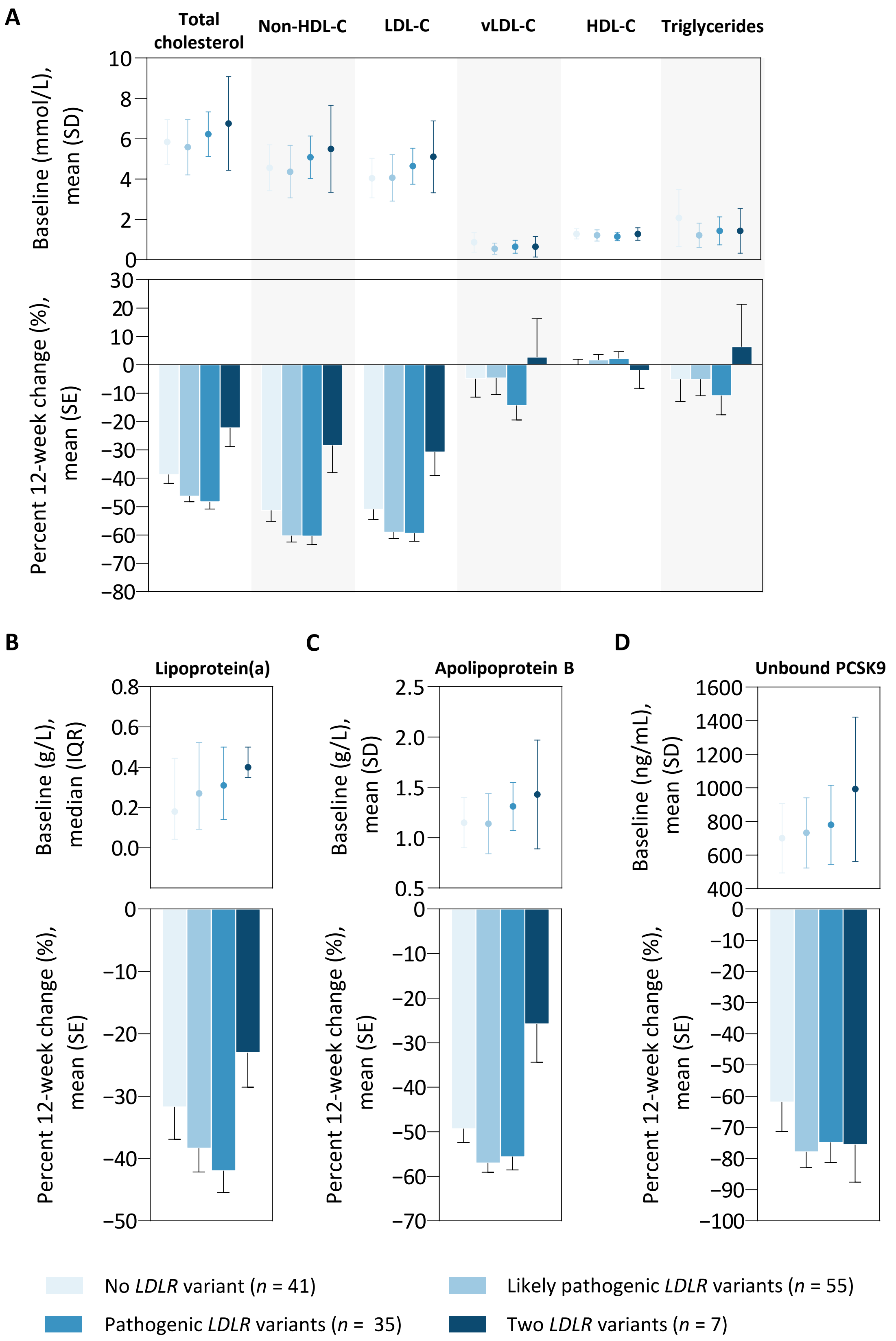

Fig. S4. Baseline and 12-week change in lipids and PCSK9 levels by *LDLR* variant pathogenicity. LDL-C = low density lipoprotein cholesterol; HDL-C = high density lipoprotein cholesterol; IQR = interquartile range; non-HDL-C = non-high density lipoprotein cholesterol; vLDL-C = very low density lipoprotein cholesterol; SD = standard deviation; SE = standard error.
