# Supplementary material for: Efficacy and safety of tafolecimab in Chinese patients with heterozygous familial hypercholesterolemia: a randomized, double-blind, placebo-controlled phase 3 trial (CREDIT-2)
Source: BMC Med. 2023 Feb 28;21:77. doi: 10.1186/s12916-023-02797-8 (PMC9976471; doi:10.1186/s12916-023-02797-8)
Supplement: Supplementary file 3 — Additional file 3: Table S1. Efficacy endpoints at week 12 assessed by treatment policy estimand. Table S2. Descriptive statistics for endpoints in lipids and unbound PCSK9 levels at week 12 and week 24. Table S3. Most commonly-reported treatment-emergent adverse events (≥5% in any group) in participants receiving tafolecimab during the 12-week double-blind treatment period and 24-week treatment period. [file 12916_2023_2797_MOESM3_ESM.pdf]

Table S1. Efficacy endpoints at week 12 assessed by treatment policy estimand.

	150 mg Q2W			450 mg Q4W		
	Tafolecimab (*n* = 52)	Placebo (*n* = 23)	ETD versus placebo	Tafolecimab (*n* = 48)	Placebo (*n* = 25)	ETD versus placebo
**LDL-C**						
Percent CFB (%)*	−55.7 (5.9)	−1.1 (6.4)	−54.6 (−66.5, −42.6)	−62.0 (5.7)	−1.3 (6.5)	−60.7 (−71.3, −50.0)
≥50% reduction*	28 (53.8)	0	54.1 (37.8, 70.3)	37 (77.1)	0	75.4 (60.8, 90.1)
<1.8 mmol/L*	25 (48.1)	1 (4.3)	44.5 (26.0, 63.1)	31 (64.6)	0	62.2 (45.8, 78.6)

Data are least squares mean (standard error) for CFB, n (%) for LDL-C target attainment rates, least squares mean (97.5%CI) for ETD.

P <0.0001 for all comparisons versus placebo.

*controlled for type I error (pairwise α = 0.025).

CFB = change from baseline; ETD = estimated treatment difference; LDL-C = low density lipoprotein cholesterol; Q2W = every 2 weeks; Q4W = every 4 weeks.

Table S2. Descriptive statistics for endpoints in lipids and unbound PCSK9 levels at week 12 and week 24.

	Tafolecimab 150 mg Q2W	Placebo 150 mg Q2W	Tafolecimab 450 mg Q4W	Placebo 450 mg Q4W
**LDL-C**				
Baseline, mmol/L	4.25 (1.115)	4.27 (0.975)	4.21 (1.143)	4.32 (1.223)
Percent CFB at week 12	−54.67 (17.257)	3.75 (18.622)	−59.16 (17.585)	1.77 (21.660)
Percent CFB at week 24	−49.61 (23.505)	−49.81 (27.545)	−56.00 (22.257)	−52.64 (22.821)
≥50% reduction from baseline to week 24	23 (50.0)	14 (60.9)	35 (72.9)	18 (78.3)
<1.8 mmol/L at week 24	21 (45.7)	9 (39.1)	28 (58.3)	12 (52.2)
**Lipoprotein(a)**				
Baseline, g/L	0.29 (0.09−0.53)	0.32 (0.10−0.46)	0.19 (0.10−0.50)	0.28 (0.12−0.49)
Percent CFB at week 12	−40.33 (24.545)	3.78 (34.134)	−39.85 (26.531)	−6.57 (21.080)
Percent CFB at week 24	−38.60 (20.415)	−37.02 (23.095)	−40.61 (26.175)	−29.83 (31.649)
**Apolipoprotein B**				
Baseline, g/L	1.20 (0.297)	1.21 (0.267)	1.19 (0.305)	1.23 (0.308)
Percent CFB at week 12	−51.09 (18.069)	8.96 (46.148)	−56.02 (16.855)	−0.39 (16.411)
Percent CFB at week 24	−47.79 (23.730)	−49.50 (24.392)	−51.79 (22.453)	−52.54 (19.906)
**non-HDL-C**				
Baseline, mmol/L	4.65 (1.284)	4.72 (1.104)	4.60 (1.332)	4.75 (1.369)
Percent CFB at week 12	−55.53 (18.933)	3.02 (18.931)	−59.74 (19.096)	0.32 (20.167)
Percent CFB at week 24	−50.14 (23.515)	−51.32 (28.399)	−56.49 (23.990)	−53.47 (25.402)
**HDL-C**				
Baseline, mmol/L	1.21 (0.254)	1.17 (0.190)	1.25 (0.283)	1.23 (0.269)
Percent CFB at week 12	2.17 (14.795)	6.28 (12.742)	0.91 (12.919)	3.29 (11.662)
Percent CFB at week 24	7.93 (17.917)	4.42 (12.875)	4.10 (12.884)	4.89 (11.887)
**Triglycerides**				
Baseline, mmol/L	1.48 (0.941)	1.66 (1.182)	1.59 (1.126)	1.54 (0.926)
Percent CFB at week 12	−5.01 (48.027)	0.53 (38.718)	−3.48 (44.278)	13.81 (57.704)
Percent CFB at week 24	−10.53 (32.209)	−17.47 (30.444)	−14.68 (28.449)	−3.80 (45.644)
**Total cholesterol**				
Baseline, mmol/L	5.86 (1.350)	5.88 (1.157)	5.85 (1.332)	5.97 (1.407)
Percent CFB at week 12	−43.16 (15.301)	3.91 (15.075)	−45.94 (14.647)	0.52 (17.050)
Percent CFB at week 24	−38.04 (19.740)	−39.54 (23.466)	−43.12 (18.969)	−40.99 (21.191)
**vLDL-C**				
Baseline, mmol/L	0.67 (0.419)	0.69 (0.352)	0.68 (0.379)	0.70 (0.413)
Percent CFB at week 12	−5.35 (44.176)	−3.87 (25.124)	−6.88 (35.142)	12.96 (59.628)
Percent CFB at week 24	−10.35 (32.659)	−11.68 (33.710)	−14.06 (30.884)	−7.09 (48.238)
**Unbound PCSK9**				
Baseline, ng/mL	707.28 (231.053)	795.74 (218.566)	785.03 (234.146)	708.71 (250.906)
Percent CFB at week 12	−68.76 (40.991)	4.41 (44.781)	−79.63 (44.632)	21.11 (37.123)
Percent CFB at week 24	−55.15 (89.597)	−66.19 (42.814)	−81.62 (28.973)	−62.78 (75.411)

Data are mean (standard interquartile range) for baseline lipoprotein(a), n (%) for LDL-C target attainment rates and mean (standard deviation) for the rest. Analysis on endpoints at week 12 and week 24 included only participants with lipids measurement at respective visit. N = 52, 23, 48 and 25 at baseline, n = 44, 22, 48 and 24 at week 12, n = 46, 23, 48 and 23 at week 24, for tafolecimab 150 mg, placebo 150 mg, tafolecimab 450 mg and placebo 450 mg, respectively.

CFB = change from baseline; LDL-C = low density lipoprotein cholesterol; HDL-C = high density lipoprotein cholesterol; non-HDL-C = non-high density lipoprotein cholesterol; Q2W = every 2 weeks; Q4W = every 4 weeks; vLDL-C = very low density lipoprotein cholesterol.

Table S3. Most commonly-reported treatment-emergent adverse events (≥5% in any group) in participants receiving tafolecimab during the 12-week double-blind treatment period and 24-week treatment period.

MedDRA system organ class<br>Preferred term, n (%)	12-week double-blind treatment		24-week treatment	
	Tafolecimab 150 mg Q2W (*n* = 52)	Tafolecimab 450 mg Q4W (*n* = 48)	Tafolecimab 150 mg Q2W (*n* = 52)	Tafolecimab 450 mg Q4W (*n* = 48)
Any TEAE	36 (69.2)	23 (47.9)	39 (75.0)	32 (66.7)
Infections and infestations	13 (25.0)	6 (12.5)	16 (30.8)	11 (22.9)
Upper respiratory tract infection	11 (21.2)	2 (4.2)	13 (25.0)	4 (8.3)
Urinary tract infection	1 (1.9)	2 (4.2)	2 (3.8)	3 (6.3)
Investigations	11 (21.2)	5 (10.4)	14 (26.9)	8 (16.7)
Blood creatine phosphokinase increased	3 (5.8)	3 (6.3)	4 (7.7)	5 (10.4)
Alanine aminotransferase increased	4 (7.7)	0	6 (11.5)	1 (2.1)
Aspartate aminotransferase increased	4 (7.7)	0	5 (9.6)	0
Musculoskeletal and connective tissue disorders	8 (15.4)	4 (8.3)	9 (17.3)	6 (12.5)
Myalgia	3 (5.8)	0	3 (5.8)	0
Skin and subcutaneous tissue disorders	5 (9.6)	2 (4.2)	6 (11.5)	2 (4.2)
Dermatitis allergic	2 (3.8)	0	3 (5.8)	1 (2.1)
Metabolism and nutrition disorders	0	2 (4.2)	5 (9.6)	3 (6.3)
Hyperuricaemia	0	0	3 (5.8)	1 (2.1)
Vascular disorders	4 (7.7)	0	4 (7.7)	1 (2.1)
Hypertension	4 (7.7)	0	4 (7.7)	1 (2.1)
Hepatobiliary disorders	2 (3.8)	1 (2.1)	3 (5.8)	1 (2.1)
Hepatic function abnormal	2 (3.8)	1 (2.1)	3 (5.8)	1 (2.1)

By MedDRA (version 24.0) system organ class and preferred term.

Q2W = every 2 weeks; Q4W = every 4 weeks.
