# Supplementary material for: Efficacy and safety of tafolecimab in Chinese patients with heterozygous familial hypercholesterolemia: a randomized, double-blind, placebo-controlled phase 3 trial (CREDIT-2)
Source: BMC Med. 2023 Feb 28;21:77. doi: 10.1186/s12916-023-02797-8 (PMC9976471; doi:10.1186/s12916-023-02797-8)
Supplement: Supplementary file 4 — Additional file 4. Narrative for the death. [file 12916_2023_2797_MOESM4_ESM.docx]

**Narrative for the death**

The subject was a 76-year-old male with a past medical history of hypertension, cataract, coronary artery disease, hyperlipidemia, renal insufficiency, and coronary artery bypass grafting. Concomitant medications included Bayaspirin, Metoprolol Tartrate Tablets, Isosorbide Mononitrate Sustained-release Tablets, Diltiazem Hydrochloride Tablets, Rosuvastatin Calcium Tablets, Nifedipine Controlled-Release Tablets, Irbesartan Hydrochlorothiazide Tablets. After signing the informed consent form on August 7, 2020, the study drug, tafolicimab 150 mg Q2W, was administered subcutaneously in the abdomen on August 11, August 24, September 07, September 21, October 09 and October 19, 2020, respectively.

On October 26, 2020, the patient was admitted due to lower abdominal pain, melena for 10 hours and hematemesis for 2.5 hours. After admission, the patient was given grade I nursing, fasting, vital sign monitoring, hemostasis, stomach protection, nutritional support. Troponin and brain natriuretic peptide gradually increased along with renal insufficiency and metabolic acidosis. Cardiology specialist consultation considered that the patient was: coronary heart disease, after coronary artery bypass grafting, acute non-ST segment elevation myocardial infarction, pump failure, and cardiac function grade II (Killip class). Later, the patient developed heart failure and was given diuresis, cardiotonic and other treatments. Later, the patient developed hemorrhagic shock due to persistent active bleeding, and was given blood transfusion again. However, during the blood transfusion, the patient suddenly lost consciousness and died after invalid rescue.

This SAE term was reported as upper gastrointestinal haemorrhage [PT: Upper gastrointestinal haemorrhage], severe. The event resulted in hospitalization or prolongation of hospitalization and led to death, and the date of event meeting the SAE was October 26, 2020. The subject discontinued medication due to the event (last dose on October 19, 2020) and the outcome of this event was death with a date of death of October 27, 2020. The investigator considered that this SAE was unrelated to the study drug according to three criteria: first, the subject had been taking Bayaspirin for a long time, which might be the cause of upper gastrointestinal bleeding; second, the subject 's poor control of hypertension aggravated bleeding; third, myocardial infarction caused by coagulation therapy.
